# Risk factors for suspected developmental delay at age 2 years in a Brazilian birth cohort

**DOI:** 10.1111/j.1365-3016.2010.01115.x

**Published:** 2010-04-08

**Authors:** Danilo R de Moura, Jaderson C Costa, Iná S Santos, Aluísio J D Barros, Alicia Matijasevich, Ricardo Halpern, Samuel Dumith, Simone Karam, Fernando C Barros

**Affiliations:** a Departamento Materno-Infantil, Universidade Federal de PelotasPelotas; b Programa de Pós-graduação em Epidemiologia, Universidade Federal de PelotasPelotas; c Programa de Pós-graduação em Medicina e Ciências da Saúde da Pontifícia Universidade Católica do Rio Grande do SulPorto Alegre; d Programa de Pós-graduação em Saúde e Comportamento, Universidade Católica de PelotasPelotas; e Departamento de Pediatria e Puericultura, Universidade Federal de Ciências da Saúde de Porto AlegrePorto Alegre; f Programa de Pós-graduação de Saúde Coletiva, Universidade Luterana do BrazilCanoas; g Área Materno Infantil – Genética Médica, Fundação Universidade de Rio GrandeRio Grande, Brasil

**Keywords:** child development, Pelotas Birth Cohort, Apgar score, maternal education, social class, maternal gestational diabetes, inter-birth interval, parenting, birthweight, gestation

## Abstract

de Moura DR, Costa JC, Santos IS, Barros AJD, Matijasevich A, Halpern R, Dumith S, Karam S, Barros FC. Risk factors for suspected developmental delay at age 2 years in a Brazilian birth cohort. *Paediatric and Perinatal Epidemiology* 2010; **24**: 211–221.

Many children are at risk of not achieving their full potential for development. Epidemiological studies have the advantage of being able to identify a number of associated factors potentially amenable to intervention. Our purpose was to identify risk factors for suspected developmental delay (SDD) at age 2 years among all children born in the city of Pelotas, Brazil, in 2004. This study was part of the 2004 Pelotas Birth Cohort. The Battelle Screening Developmental Inventory (BSDI) was administered to cohort children at age 2 years. A hierarchical model of determination for SDD with confounder adjustment was built including maternal sociodemographic, reproductive and gestational characteristics, as well as child and environmental characteristics. Multivariable analysis was carried out using Poisson regression. Prevalence ratios (PR) and 95% confidence intervals [95% CI] were calculated.

In the results, 3.3% of the 3869 children studied screened positive for SDD. After confounder control, children more likely to show SDD were: those with positive BSDI at age 12 months (PR = 5.51 [3.59, 8.47]); with 5-min Apgar <7 (PR = 3.52 [1.70, 7.27]); with mothers who had <4 years of schooling (PR = 3.35 [1.98, 5.66]); from social classes D and E (PR = 3.00 [1.45, 6.19]); with a history of gestational diabetes (PR = 2.77 [1.34, 5.75]); born <24 months after the last sibling (PR = 2.46 [1.42, 4.27]); were not told child stories in the preceding week (PR 2.28 [1.43, 3.63]); did not have children's literature at home (PR = 2.08 [1.27, 3.39]); with low birthweight (PR = 1.75 [1.00, 3.07]); were born preterm (PR = 1.74 [1.07, 2.81]); with <6 antenatal care appointments (PR = 1.70 [1.07, 2.68]); with history of hospitalisation (PR = 1.65 [1.09, 2.50]); and of male sex (PR = 1.43 [1.00, 2.04]). These risk factors may constitute potential targets for intervention by public policies and may provide help to paediatricians in preventing developmental delay.

## Introduction

It is estimated that, worldwide, 200 million children under 5 years of age are at risk of not fully achieving their developmental potential.^1^ Human development is shaped by a dynamic and continuous interaction between biology and experience.^2^ Individual developmental pathways throughout the life cycle are influenced by interactions among risk factors that, on one side, increase the probability of a poor outcome and on the other side are protective factors that increase the probability of a positive outcome.^3^ The American Academy of Pediatrics recommends that surveillance and monitoring instruments for developmental delay are systematically used in order to identify children at risk and to introduce stimulation measures in a timely manner. There is evidence that early intervention can reduce the risk of developmental delay in older children.^4^,^5^ Stimulation can lead to not only functional, but also structural modification of the brain.^6^

Given the multifactorial character of developmental delay, epidemiological studies have the advantage of being able to identify a large number of associated factors potentially amenable to intervention. Most of the research on child development comes from developed countries; few studies are conducted in under-developed settings.^7^ The present study aimed to identify risk factors for suspected developmental delay (SDD) at age 2 years among all children born in the city of Pelotas, Brazil, in 2004.

## Methodology

### Population and study design

The population of the present study was a cohort of children born in 2004 in Pelotas, a city with a population of 340 thousand inhabitants, located near the Southern border of Brazil with Uruguay and Argentina. The population originates mostly from European (Portuguese, Spanish and German) and African immigrants, and native Americans. The main economic activities are agriculture (mostly rice and cattle raising), commerce and education.

All livebirths (*n* = 4231) from mothers living in the urban area of Pelotas and in the Jardim America neighbourhood (which belongs to the neighbouring municipality of Capão do Leão), were included in the study. Children were included in the cohort at birth, during their stay at the hospital of delivery, and were followed up at ages 3, 12 and 24 months. A total of 3869 children were visited at home at 24 months of age.

### The outcome and the Battelle Screening Developmental Inventory

The outcome was investigated using the Battelle Screening Developmental Inventory (BSDI),^8^ which was administered to the children at home, within ±30 days of their second birthday, by a trained female interviewer. The BSDI test consists of 96 items with three administration formats: structured administration, observation, and interviews with parents or other sources.^9^ The BSDI was translated into Portuguese from the Spanish version, and the resulting text was pre-tested with interviewers for clarity and revised by the investigators for fidelity to the original meaning. The test was performed by interviewers who were trained by a paediatrician who was specialised in child development. The test evaluates child development in five domains: personal-social, adaptive, motor, communication and cognitive development. The sum of the scores for each domain generates a total score. Individual results were classified as either ‘normal’ or ‘suspected delay’ according to a cut-off point of −1 SD in the table of total scores of the reference population.

### The explanatory variables

The explanatory variables were obtained from questionnaires administered to mothers in the hospital at the time of the child's birth (perinatal), and on the occasion of the 12- and 24-month follow-up visits. To identify the variables that were independently associated with SDD a conceptual framework was used.^10^ This framework was organised in levels that were then used in the multivariable analyses. Family income and mother's years of schooling years are at the most distal level, followed by maternal and gestational characteristics. These variables may determine the occurrence of perinatal events, maternal and child morbidity which, in turn, may influence the child's development. Some of the proximal factors linked to the caregiver's quality of care giving, such as telling the child stories and watching television, may also have an effect on the child's development. Although this conceptual hierarchical framework is derived from the field of child health in less-developed countries, the general principles also apply to a number of other health problems both in developed and less-developed countries.^10^ Furthermore, the same hierarchical framework has been used in other birth cohort studies to assess risk factors for SDD.^11^,^12^

During the perinatal interview, information on maternal sociodemographic, reproductive and gestational characteristics was obtained using structured questionnaires.

### The first level: maternal sociodemographic variables

Mother's age was recorded in completed years. Skin colour was self-reported, and classified as white, black or mixed. For economic classification, the Brazilian Criterion for Economic Classification, of the Brazilian Association of Market Research Companies was used. Economic class status was divided into five groups: A (wealthiest), B, C, D and E (poorest).^13^,^14^ Maternal schooling corresponds to completed years of formal education.

### The second level: maternal reproductive variables

Birth spacing corresponded to the time interval between the birth of the current and immediately preceding children, classified as primipara, <24 and ≥24 months. The number of antenatal care appointments was obtained from the mother's pregnancy card or, if unavailable, was directly reported by the mother.

### The third level: gestational variables

History of anaemia, diabetes mellitus and arterial hypertension during pregnancy were considered positive only when the mother declared having been diagnosed with one of these conditions by a physician. Information on smoking during pregnancy was also collected, considering as smokers those mothers who reported smoking at least one cigarette, on a daily basis, during any trimester of pregnancy.

### The fourth level: perinatal variables

Data on the newborn baby included mode of delivery (vaginal or caesarean section), 5-min Apgar, weight and gestational age. Newborns weighing <2500 g were considered as low birthweight (LBW). For assessing gestational age at birth an algorithm proposed by the National Center for Health Statistics^15^ was applied. The estimated age was based on the last menstrual period whenever it was consistent with birthweight, length and head circumference, based on the normal curves for these parameters for each week of gestational age.^16^ In case the last menstrual period-based gestational age was unknown or inconsistent, we adopted the clinical maturity estimate based on the Dubowitz method, which was performed on all newborns.^17^ Babies were categorised in terms of weight-for-gestational age according to the Williams criterion,^16^ into adequate, small or large-for-gestational-age at birth.

### The fifth level: child nutritional variables and mother–child morbidity

The result of BSDI evaluation of SDD administered to the subject at home within ±30 days of his or her first birthday was extracted from the 12-month follow-up. Variables pertaining to nutrition, mother and child morbidity, and environmental stimuli were obtained at the 24-month follow-up. Duration of breast feeding was categorised into five groups: <1, 1–3, 4–6, 7–12 and ≥13 months. Length-for-age deficit and excess weight-for-length were defined according to World Health Organization standards, using cut-off points corresponding to −2.00 and +2.00 SD Z scores, respectively.^18^ Information on history of febrile and non-febrile seizures was directly reported by the mother. All hospital admissions during the first 2 years of life were recorded. Maternal depression at 24 months post-delivery was assessed using the Edinburgh Postnatal Depression Scale (EPDS).^19^ The EPDS was validated with a sample of mothers from the 2004 Pelotas Birth Cohort, showing a sensitivity of 59.5% and a specificity of 88.4% for diagnosis of maternal depression (at the cut-off point ≥13).^20^

### The sixth level: variables related to stimulation

Environmental stimuli studied included presence of children's books or comic books in the household, whether the child had been exposed to stories in the last week (as long as told by a person or recording, but excluding television or video programmes), and time spent watching television.

### Quality control

All interviews were carried out by trained interviewers. Interviewers went through retraining sessions every 2 months aimed at maintaining a high level of standardisation. Also for quality control purposes, 5% of all interviews were repeated using an abbreviated version of the questionnaire, and 40% of mothers were contacted by telephone to ascertain that interviews were being carried out adequately and in full. Data were entered twice, by two independent technicians, using Epi Info software. v. 6.4.

### Statistical analysis and the hierarchical model

**Figure 1:**
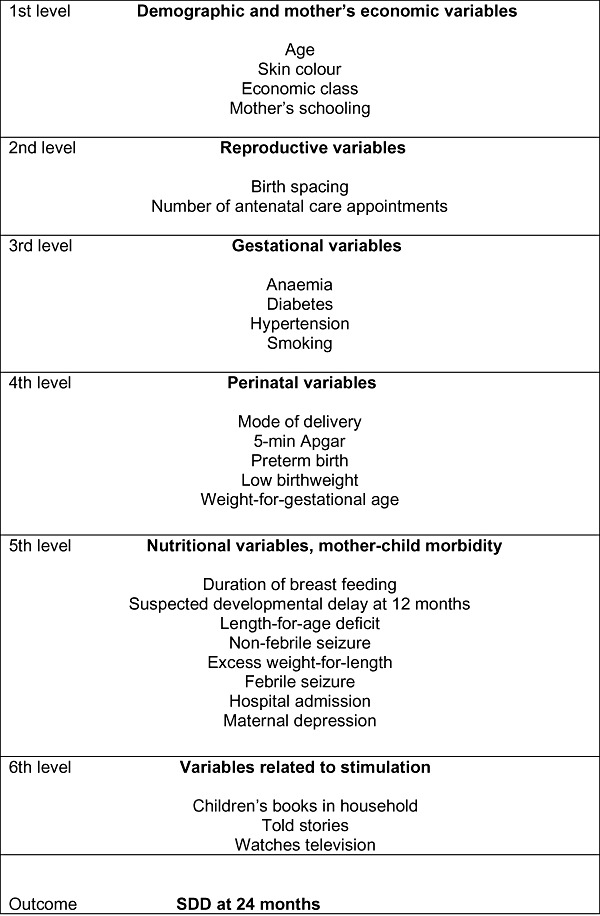
Conceptual hierarchical model for the causality of suspected developmental delay (SDD) at age 24 months.

For analysis purposes, a hierarchical model of determination, based on the conceptual framework, was constructed. This model allows quantifying the contribution of each level to SDD (Fig. 1). Confounder control was carried out for variables in the same level or immediately superior levels. Variables with *P* values below 0.20 were maintained in the final multivariable analysis model. In both univariable and multivariable analysis, the associations between explanatory variables and the outcome were assessed using the Wald test, with a 5% significance threshold. Prevalence ratios (PR) and 95% confidence intervals [CI] were also calculated. Multivariable analyses were carried out using Poisson regression, which provides more reliable estimates of the relative risk than logistic regression when analysing binary outcomes from cross-sectional studies.^21^ Data consistency assessment, variable edition and statistical analyses were carried out using STATA software, v. 8.

### Ethics

The 2004 Pelotas Birth Cohort Study was approved by the Research Ethics Committee of the University of Pelotas School of Medicine and by the Ethics Committee of the World Health Organization (Geneva). Prior to providing written consent, a term of informed consent explaining the study's goals and procedures was read out to mothers. Confidentiality of collected information, voluntary participation, and the possibility of leaving the study without any consequences for either mother or child were ensured to all participants.

## Results

**Figure 2:**
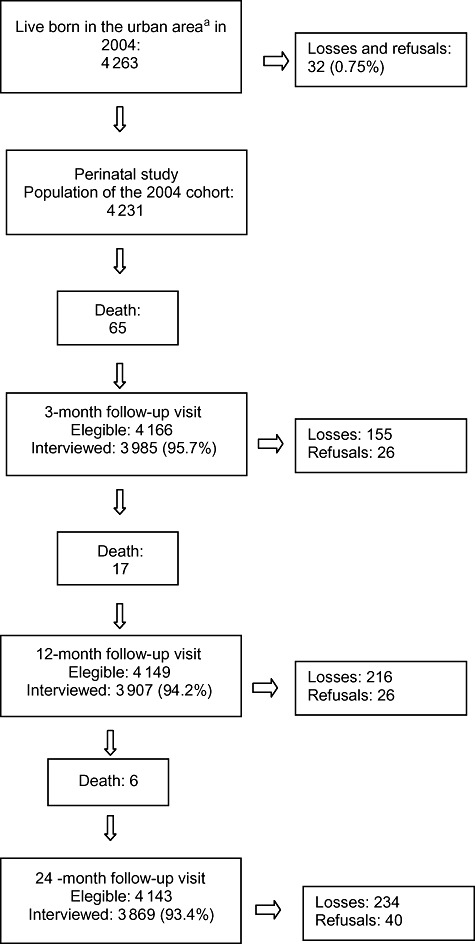
Diagram indicating the number of births, deaths, losses and refusals in the Pelotas Birth Cohort, 2004. ^a^Includes children born of mothers living in the Jardim América neighbourhood, which is contiguous to the Pelotas urban area but belongs to the municipality of Capão do Leão.

Figure 2 shows the numbers of children born in Pelotas in 2004, followed up in the subsequent visits, and losses and refusals up to 24 months. Over 90% of children were traced in all follow-ups. Table 1 describes the characteristics of mothers and 3869 children included in the current study. Table 1 also presents the prevalence of SDD according to the explanatory variables, as well as the results of univariable and multivariable adjusted analyses.

**Table 1:** Prevalence of positive Battelle Screening Developmental Inventory (BSDI) and crude and adjusted prevalence ratios (PR) according to explanatory variables: 2004 Pelotas Birth Cohort, Pelotas, Brazil, 2008

				Univariable analysis		Multivariable analysis	
					
Level	Variables	*n*	Positive BSDI (%)	PR [95% CI]	*P*	PR^a^[95% CI]	*P*
	Child sex				0.058		0.053
	Male	2011	3.8	1.40 [0.99, 2.00]		1.43 [1.00, 2.0])	
	Female	1858	2.7	1.00 Reference		1.00 Reference	
1	Mother's skin colour				0.090		0.972^b^
	White	2372	2.8	1.00 Reference		1.00 Reference	
	Mixed	782	4.1	1.47 [0.97, 2.22]		1.04 [0.68, 1.59]	
	Black	633	4.1	1.48 [0.94, 2.30]		0.98 [0.61, 1.55]	
1	Mother's age (years)				0.695		0.470^b^
	≤19	722	3.3	1.06 [0.69, 1.66]		0.83 [0.52, 1.33]	
	20–35	2734	3.1	1.00 Reference		1.00 Reference	
	≥36	411	3.8	1.25 [0.74, 2.11]		1.26 [0.74, 2.17]	
1	Mother's schooling (years)				<0.001		<0.001^c^
	0–4	588	7.3	5.29 [3.24, 8.64]		3.35 [1.98, 5.66]	
	5–8	1504	3.6	2.64 [1.64, 4.25]		1.81 [1.12, 2.94]	
	≥9	1737	1.3	1.00 Reference		1.00 Reference	
1	Economic class				<0.001		<0.001^c^
	A/B	784	1.0	1.00 Reference		1.00 Reference	
	C	1599	2.3	2.21 [1.03, 4.73]		1.51 [0.71, 3.21]	
	D/E	1387	5.6	5.44 [2.64, 11.20]		3.00 [1.45, 6.19]	
2	Birth spacing				<0.001		0.005^b^
	<24 months	319	8.5	4.72 [2.81, 7.94]		2.46 [1.42, 4.27]	
	≥24 months	1742	3.2	1.76 [1.12, 2.78]		1.29 [0.82, 2.04]	
	Primiparae	1507	1.8	1.00 Reference		1.00 Reference	
2	No. antenatal care appointments				<0.001		0.023^b^
	0–5	661	7.9	3.43 [2.42, 4.86]		1.70 [1.07, 2.68]	
	≥6	3048	2.3	1.00 Reference		1.00 Reference	
3	Gestational hypertension				0.095		0.238^b^
	No	2951	2.9	1.00 Reference		1.00 Reference	
	Yes	910	4.1	1.39 [0.94, 2.01]		1.28 [0.85, 1.93]	
3	Gestational diabetes				0.222		0.006^b^
	No	3751	3.2	1.00 Reference		1.00 Reference	
	Yes	115	5.2	1.64 [0.73, 3.65]		2.77 [1.34, 5.75]	
3	Gestational anaemia				0.262		0.083^b^
	No	1286	2.9	1.00 Reference		1.00 Reference	
	Yes	2556	3.5	1.24 [0.84, 1.82]		1.48 [0.95, 2.29]	
3	Smoking during pregnancy				<0.001		0.213^b^
	No	2812	2.6	1.00 Reference		1.00 Reference	
	Yes	1057	5.0	1.93 [1.36, 2.73]		1.29 [0.86, 1.92]	
4	Preterm birth				<0.001		0.025^b^
	No	3332	2.5	1.00 Reference		1.00 Reference	
	Yes	532	7.7	3.13 [2.18, 4.51]		1.74 [1.07, 2.81]	
4	Mode of delivery				0.192		0.516^b^
	Vaginal	2113	3.6	1.00 Reference		1.00 Reference	
	C-section	1756	2.9	0.79 [0.55, 1.12]		1.14 [0.77, 1.69]	
4	Low birthweight				<0.001		0.049^b^
	No	3520	2.7	1.00 Reference		1.00 Reference	
	Yes	348	8.6	3.19 [2.15, 4.74]		1.75 [1.00, 3.07]	
4	Apgar 5				<0.001		0.001^b^
	0–6	62	17.7	5.89 [3.35, 10.38]		3.52 [1.70, 7.27]	
	7–10	3787	3.1	1.00 Reference		1.00 Reference	
4	Weight-for-gestational age				0.014		0.543^b^
	Small	480	5.2	1.87 [1.21, 2.88]		1.28 [0.74, 2.22]	
	Adequate	3116	2.8	1.00 Reference		1.00 Reference	
	Large	269	4.1	1.47 [0.79, 2.71]		1.29 [0.66, 2.51]	
5	Z-scores length-for-age				<0.001		0.191^b^
	<−2.00	190	13.7	5.02 [3.35, 7.54]		1.48 [0.82, 2.68]	
	≥−2.00	3669	2.7	1.00 Reference		1.00 Reference	
5	Z-scores weight-for-age				0.095		0.178^b^
	<2.00	3586	3.4	1.00 Reference		1.00 Reference	
	≥2.00	273	1.5	0.43 [0.16, 1.16]		0.49 [0.18, 1.38]	
5	Duration of breast feeding (months)				<0.001		0.355^c^
	<1	426	6.1	2.64 [1.58, 4.41]		1.41 [0.78, 2.57]	
	1–3	898	4.6	1.97 [1.24, 3.14]		1.12 [0.65, 1.95]	
	4–6	589	2.0	0.88 [0.45, 1.71]		1.23 [0.63, 2.39]	
	7–12	653	2.5	1.06 [0.58, 1.93]		1.17 [0.61, 2.27]	
	≥13	1297	2.3	1.00 Reference		1.00 Reference	
5	Hospital admission				<0.001		0.018^b^
	No	2823	2.09	1.00 Reference		1.00 Reference	
	Yes	962	6.55	3.13 [2.21, 4.43]		1.65 [1.09, 2.50]	
5	Non-febrile seizure				<0.001		0.061^b^
	No	3803	0.3	1.00 Reference		1.00 Reference	
	Yes	65	18.5	6.15 [3.58, 10.59]		2.22 [0.97, 5.10]	
5	Febrile seizure				0.673		0.938^b^
	No	3741	3.2	1.00 Reference		1.00 Reference	
	Yes	128	3.9	1.20 [0.50, 2.90]		1.03 [0.49, 2.18]	
5	Positive Battelle at 12 months				<0.001		<0.001^b^
	No	3411	1.9	1.00 Reference		1.00 Reference	
	Yes	372	15.6	8.44 [6.01, 11.86]		5.51 [3.59, 8.47]	
5	Maternal depression				0.030		0.896^b^
	No	3209	2.9	1.00 Reference		1.00 Reference	
	Yes	612	4.6	1.58 [1.04, 2.39]		0.97 [0.60, 1.56]	
6	Presence of children's books				<0.001		0.003^b^
	No	1721	5.8	4.80 [3.13, 7.35]		2.08 [1.27, 3.39]	
	Yes	2146	1.2	1.00 Reference		1.00 Reference	
6	Child told stories				<0.001		<0.001^b^
	No	1813	5.2	3.46 [2.32, 5.16]		2.28 [1.43, 3.63]	
	Yes	2047	1.5	1.00 Reference		1.00 Reference	
6	Child watches television				0.001		0.685^b^
	No	642	5.6	1.00 Reference		1.00 Reference	
	≤2 h	2170	2.7	0.48 [0.32, 0.72]		0.81 [0.50, 1.31]	
	>2 h	949	3.1	0.55 [0.34, 0.88]		0.85 [0.49, 1.49]	

**–**	Total	3869	3.3	**–**	**–**	**–**	**–**

a PRs shown for the adjusted analysis are adjusted only for variables presenting a *P* value <0.20 in the same or in the upper levels of the conceptual model.

b Wald test for heterogeneity.

c Wald test for linear trend.

Suspected developmental delay was evaluated in 3869 children located at age 24 months, yielding a prevalence of 3.3% [95% CI 2.7, 3.8], accounting for 128 children. After adjustment for confounders, the variables listed below showed statistically significant associations with SDD.

### Maternal sociodemographic variables

In the first level, boys presented a risk of SDD 43% higher than girls. Both maternal schooling and economic class were inversely associated with SDD. Children of mothers with between 0 and 4 years of schooling were over three times more likely to show SDD than those of mothers with ≥9 years of schooling. For the children of mothers with 5–9 years of schooling, prevalence was 81% greater than that of children in the ≥9 years group. In relation to classes A and B, taken as a reference, risk of SDD was 1.5- and threefold higher among children from classes C and D/E, respectively.

### Maternal reproductive variables

Among reproductive variables, after allowing for maternal schooling, economic class and child's sex, a higher PR was found for birth spacing shorter than 24 months (PR = 2.46; [1.42, 4.27]) and for <6 antenatal care appointments (PR = 1.70; [1.07, 2.68]).

### Gestational variables

At the level of gestational variables, children born to diabetic mothers presented an almost threefold higher PR for SDD than their controls (PR = 2.77; [1.34, 5.75]). Variables from the third level were adjusted for each other and for child's sex, mother's schooling, economic class, birth spacing and number of antenatal care appointments.

### Perinatal variables

At the fourth level (perinatal variables), preterm birth (PR = 1.74; [1.07, 2.81]), LBW (PR = 1.14; [0.77, 1.69]) and 5-min Apgar <7 (PR = 3.52; [1.70, 7.27]) were associated with greater likelihood of SDD. The multivariable model included all the potential confounders for variables of the third level in addition to maternal diabetes and anaemia during pregnancy.

### Child nutritional variables and mother–child morbidity

At the level of variables related to child morbidity (adjusting for preterm birth, LBW, 5′Apgar, height-for-age z-score, weight-for-height z-score and history of non-febrile seizures, besides the potential confounders of the previous levels), children with history of hospital admission (PR = 1.65; [1.09, 2.50]) and positive BSDI at 12 months (PR = 5.51; [3.59, 8.47]) presented greater PRs for SDD.

### Variables related to stimulation

In the final level – factors associated with stimulation – after allowing for the above potential confounders, children whose households lacked children's books (PR = 2.08; [1.7, 3.39]) and who had not been told stories during the preceding week (PR = 2.28; [1.43, 3.63]) showed over twofold higher PRs of SDD than their counterparts.

## Discussion

The current study found that, after adjustment for potential confounders, child's sex, economic class, mother's schooling, birth spacing, gestational diabetes, preterm birth, LBW, 5-min Apgar, hospital admission, SDD at 12 months, children's books at home and child told stories were associated with SDD at 24 months of age.

Strengths of this study include its cohort design, which allows for temporality of the association between exposures and the outcome. In terms of external validity, socio-economic and contextual characteristics of Pelotas are likely to represent the reality of most of the middle-sized cities from middle-income countries. This study also has some limitations. First, the BSDI has not been previously validated in a Brazilian population. Thus, it is likely that the observed accuracy with the population in which it was first tested^8^ does not correspond to the one when applied to Brazilian children. Second, information on maternal and child characteristics was gathered by maternal recall, and thus may suffer from information bias.

The association between economic class and SDD has also been reported by Pilz and Schermann in a study carried out in another Brazilian municipality.^22^ The finding that mother's schooling is independently associated with SDD is also in agreement with results from other authors.^23^,^24^ Associations with birth spacing of <24 months and with <6 antenatal care appointments were also reported in another study carried out in the same Brazilian state.^22^

In univariable analysis, diabetes was not significantly associated with the outcome. This association was clouded by a likely negative confounding effect of social class and maternal schooling. Mothers from more affluent social classes and with greater schooling (conditions that are shown to be protective against SDD) had a larger proportion of diabetics. This association has high biological plausibility, given that children of diabetic mothers are subject to metabolic events in the neonatal period that, depending on the quality of care, may lead to neurological lesions with sequelae that may affect child development.

In the present population, 27% of mothers smoked during pregnancy; however, this exposure was not associated with SDD. Another study investigating smoking during pregnancy and its effects on cognitive development and children's skills also failed to detect an association between these variables after control for confounders.^23^ The lack of association between smoking during pregnancy and SDD may be due to the way the variable was constructed. Smoking was a dichotomic variable (yes or no), independently of the intensity of smoking. A recent study has found an association with intellectual disabilities when mothers smoked 20 or more cigarettes per day.^25^ The higher PRs observed between preterm and LBW births with SDD in comparison, respectively, with full-term and non-LBW births are in agreement with the results of several other studies.^26^–^30^ LBW and preterm birth were also shown to be independently associated with specific delays in motor and social development.^23^ Lack of association between mode of delivery and SDD has been reported by other authors.^31^,^32^

Being either small or large for gestational age was not associated with positive screening for SDD, as previously shown by another author.^33^ However, being small for gestational age appeared as a risk factor for delays in skill acquisition in another study.^34^

Children with 5′Apgar <7 were three times more likely to show SDD at 24 months. The biological mechanism underlying this finding is unclear, given that no single parameter (Apgar, cord pH or heartbeat frequency) can be used as a synonym for asphyxia. Other authors have shown that sequelae are more closely related to one of the three stages of ischaemic hypoxic encephalopathy than to any specific indicator.^35^–^37^

In the analysis of nutritional determinants, neither maternal malnutrition nor obesity was associated with SDD. Several studies have indicated breast feeding as a promoting factor for child development.^38^–^42^ Univariable analysis of the current data showed that the longer the child was breast fed, the lower the prevalence of SDD; however, the statistical significance of this association was lost after adjustment for confounders. Another study also failed to detect an association between duration of breast feeding and child development at 12 months.^33^ However, given the moderate specificity of BSDI, it is possible that children with more severe delays were detected to the expense of milder cases. It is therefore possible that children with mild SDD, usually related to insufficient stimulation, would benefit the most from breast feeding. It is also possible that the benefits of breast feeding in terms of cognitive skills may emerge only at a later age. Recent studies have demonstrated that the interaction between individual genetic characteristics and environmental factors can play a role in child intelligence.^43^ It could also be possible that maternal recall of breast-feeding duration was not accurate. However, a review study reported that maternal recall of breast-feeding duration is reliable, mainly when information is collected with a time interval lower than 3 years.^44^ In the 2004 Pelotas Birth Cohort, data on breast feeding was obtained from follow-ups at 3, 12 and 24 months of age, very close to the moment of weaning.

History of hospital admission has been shown to be a risk factor for SDD in studies of preterm and LBW babies.^45^,^46^ In the present study, this variable was associated with the outcome even after control for confounders. The frequency of children hospitalised at least once during their first year of life was 19.2%.

Children with a history of non-febrile convulsions in the first 2 years of life were 2.5 times more likely to show SDD; however, in the multivariable analysis, the inclusion of the variable SDD at 12 months to the model turned this association statistically non-significant. Another study reported non-association between non-febrile convulsions in the first 2 years and developmental delay.^47^ The present study also provides support for the benign character of febrile convulsions, which were not associated with the outcome in either univariable or multivariable analysis.

In the current study, prevalence of SDD was 3.3% at age 24 months, whereas prevalence of SDD at 12 months was about 10%, showing that the trajectory is to improve with time across all domains of development. No specific interventions to manage developmental issues were delivered to children that were positive at the 12-month screening. These children continued to receive the usual paediatric care from the health system of the city. From children who screened positive at 12 months, 15.6% [95% CI 11.9, 19.3] remained positive for SDD at 24 months. Further analyses showed that the following were prognostic factors for persistence of SDD: Apgar 5′ <7, low socio-economic level, intergestational interval <24 months, breast-feeding duration ≤6 months and not having been told stories in the previous 2 weeks (unpublished data).^48^

Among environmental variables related to stimulation, having been told stories in the previous week and presence of children's literature (books, comic books) in the household were found to be highly protective, even after confounder control. This finding is potentially relevant for intervention purposes, since children's literature was absent from almost half of the households. However, this may be due to reverse causality, i.e. children without developmental delay may request more attention from their parents, including asking them to tell them stories, than children with developmental delay.

As to exposure to television, the American Academy of Pediatrics recommended in 2001 that children under the age of 2 years not watch television.^49^ Several studies have shown a negative effect of duration of exposure to television on child cognitive development.^50^,^51^ In the current study no association was found between television viewing and child development. However, a distinction must be made between programmes created for adults and those created for children. It is possible that the content of the television programmes watched may determine what influence television has on child development.^52^

Another analysis of the 2004 Pelotas Birth Cohort, aiming to examine child development at 2 years of age and its psychosocial determinants, employed five markers of cognitive stimulation (whether someone read or told a story to the child; whether the child went to a park or playground; whether the child went to some other people's houses; whether the child watched TV; and whether the child had a story book) that were recorded and summed in a score ranging from 0 to 5.^53^ This analysis found that child development was strongly associated with socio-economic position, maternal schooling and stimulation. Having been told a story and owning a book were the least frequent markers among children with low scores.

## Conclusion

Identifying risk factors is important for establishing policies for prevention of developmental delay. Local studies aimed at identifying risk factors play an important role in the establishment of intervention strategies.^1^ The present results suggest certain public policies for prevention of developmental delay. These include increasing the number of antenatal care appointments, increasing spacing between pregnancies, reducing preterm delivery and improving the quality of care at delivery. Encouraging the practice of telling or reading stories and improving the availability of children's literature in the household are a feasible, perhaps less costly intervention, that deserve to be formally tested to prevent developmental delay.
